# Effectiveness of mHealth interventions for patients with diabetes: An overview of systematic reviews

**DOI:** 10.1371/journal.pone.0173160

**Published:** 2017-03-01

**Authors:** Spyros Kitsiou, Guy Paré, Mirou Jaana, Ben Gerber

**Affiliations:** 1 Department of Biomedical and Health Information Sciences, College of Applied Health Sciences, University of Illinois at Chicago, Chicago, United States of America; 2 Research Chair in Digital Health, Department of Information Technologies, HEC Montréal, Montréal, Canada; 3 Telfer School of Management, University of Ottawa, Ottawa, Canada; 4 Academic Internal Medicine and Geriatrics, College of Medicine, University of Illinois at Chicago, Chicago, United States of America; University of Rochester, UNITED STATES

## Abstract

**Background:**

Diabetes is a common chronic disease that places an unprecedented strain on health care systems worldwide. Mobile health technologies such as smartphones, mobile applications, and wearable devices, known as mHealth, offer significant and innovative opportunities for improving patient to provider communication and self-management of diabetes.

**Objective:**

The purpose of this overview is to critically appraise and consolidate evidence from multiple systematic reviews on the effectiveness of mHealth interventions for patients with diabetes to inform policy makers, practitioners, and researchers.

**Methods:**

A comprehensive search on multiple databases was performed to identify relevant systematic reviews published between January 1996 and December 2015. Two authors independently selected reviews, extracted data, and assessed the methodological quality of included reviews using AMSTAR.

**Results:**

Fifteen systematic reviews published between 2008 and 2014 were eligible for inclusion. The quality of the reviews varied considerably and most of them had important methodological limitations. Focusing on systematic reviews that offered the most direct evidence, this overview demonstrates that on average, mHealth interventions improve glycemic control (HbA1c) compared to standard care or other non-mHealth approaches by as much as 0.8% for patients with type 2 diabetes and 0.3% for patients with type 1 diabetes, at least in the short-term (≤12 months). However, limitations in the overall quality of evidence suggest that further research will likely have an important impact in these estimates of effect.

**Conclusions:**

Findings are consistent with clinically relevant improvements, particularly with respect to patients with type 2 diabetes. Similar to home telemonitoring, mHealth interventions represent a promising approach for self-management of diabetes.

## Introduction

Diabetes is a complex chronic disease that afflicts millions of individuals worldwide, resulting in significant morbidity, mortality, and health care resources utilization [1–6]. With projected increases in the prevalence of diabetes and costs arising from the long-term complications incurred by this condition (e.g. cardiovascular disease, strokes, retinopathy, nephropathy, and amputations), policy makers and health care providers continue to focus on identifying self-management interventions that improve diabetes care and patient related outcomes. Prior research has shown that diabetes self-management education and support improve hemoglobin A1c (HbA1c) levels by as much as 1%, reduce the risk of developing debilitating and life‐threatening complications, and have a positive effect on other psychosocial and behavioral aspects of diabetes [7]. However, providing optimal care for patients with diabetes remains a challenge for healthcare systems and providers. Patients often encounter various barriers in adhering to self-management programs due to lack of knowledge and understanding of self-care activities, lack of individualized and coordinated care, inconvenient and costly education sessions, and poor patient-provider communication [8].

Mobile technologies such as cell phones/smartphones, handheld tablets, and other wireless devices known as mHealth, offer new and exciting opportunities for addressing some of these challenges by enabling remote patient monitoring and delivery of clinical advice through a wide-range of functions (e.g. text messaging, web browsing, email, and videos). Mobile phones are now omnipresent with worldwide usage rates nearing 100% (96% globally; 128% in developed countries; and 89% in developing countries) [9]. The ubiquitous nature of mobile phones coupled with their constantly evolving processing and connectivity power create opportunities for new and innovative approaches to support blood glucose and diet monitoring, measurement of daily physical activity, education, and other activities that can facilitate diabetes self-management and enhance patient-provider communication [10, 11].

Current interest in mHealth interventions to improve management of diabetes has led to a plethora of empirical studies [12]. Healthcare providers, researchers, and policy makers who have an interest in the effects of mHealth for diabetes management are inundated with vast amounts of information from numerous clinical trials and observational studies. Evidence syntheses, and in particular systematic reviews (SRs) partly address the problem of information overload by pooling together and collating all the available evidence from multiple trials. However, practitioners often find it difficult to reliably retrieve and keep up to date with the growing volume of SRs that is published in a variety of formats and sources. Furthermore, as with all types of research, available SRs may vary in quality and their findings can potentially be flawed due to methodological issues and risks of bias. These flaws can cause a SR to be biased and potentially misleading for decision-making. Prior research in the area of home telemonitoring for chronic disease management has shown that not all SRs are truly systematic and their methodological quality varies significantly [13, 14]. Hence, critical appraisal and synthesis of this evidence is needed to provide reliable and accessible information to clinicians and decision makers. Overviews of SRs (also known as umbrella reviews) [15] are an efficient way to critically appraise prior reviews and gather the best available evidence in a single source to provide broad, cumulative statements that summarize the current evidence and knowledge on the effectiveness of interventions [16,17]. Such overviews are helpful and can serve as starting points for decision makers to unpack the evidence towards finding solutions to improving practice and identify areas where new research is needed [16].

This overview of SRs aims to collect, appraise, and synthesize evidence from multiple SRs and meta-analyses on the effectiveness of mHealth interventions for patients with diabetes, in order to provide researchers, policy makers and practitioners with the evidence they need to make informed decisions. It also aims to identify research gaps that need to be addressed and develop a series of recommendations for improving the quality of future reviews and studies in this area.

## Methods

### Search strategy

We searched MEDLINE, EMBASE, CINAHL, the Cochrane Database of Systematic Reviews, Database of Abstracts of Reviews of Effect (DARE), and Health Technology Assessment (HTA) databases, to identify SRs of mHealth interventions for diabetes published between January 1, 1996 and December 31, 2015. Searches were supplemented by hand-searching reference lists of relevant SRs. The search strategy combined multiple keywords (e.g. mHealth, telehealth, and text-messaging) with subject heading terms (e.g. diabetes mellitus, cellular phone, and telemedicine) and specialized clinical queries for SRs (e.g. systematic[sb] in MEDLINE). No language or publication type restrictions were applied. The full search strategy for all databases is available in S1 Appendix.

### Screening and selection of systematic reviews

Two reviewers (SK and GP) independently examined the titles and abstracts of all the identified references. Articles eligible for inclusion were SRs (as identified in the PRISMA statement [18]) examining the effectiveness of interventions involving mobile technologies such as cellular phones, personal digital assistants (PDAs), tablet PCs (e.g. iPads), and other wireless devices that can enable remote patient monitoring and delivery of clinical advice through a wide range of functions and applications (e.g. text messaging, internet, email, and videos) for self-management of type 1 diabetes (T1D) or type 2 diabetes (T2D). References that clearly did not meet all of the criteria were excluded. The full-text article of all references that appeared to be relevant was retrieved and independently assessed by three reviewers in groups of two (SK, GP, and MJ). Disagreements were resolved through discussion in team meetings.

### Assessment of methodological quality

Two reviewers (SK & GP) independently assessed the methodological quality of the included SRs using AMSTAR [19]. AMSTAR is a validated instrument that uses 11 items to assess the degree to which the design and execution of a SR are methodologically sound and unbiased. Each item is categorized into a standardized set of four possible responses: “yes”, “no”, “can’t answer” or “not applicable”. The items relate to an a priori design, study selection and data extraction, comprehensiveness of the search strategy, search of grey literature for eligible studies, reporting of included and excluded studies, presentation of study characteristics, conduct of risk of bias assessment, appropriateness of methods used to synthesize study findings, formulation of conclusions taking into consideration the overall quality of evidence, publication bias and conflict of interest assessments. To ensure consistency of assessment between the two assessors, we developed decision support rules for scoring each criterion (S2 Appendix) [14]. Based on the results of the critical appraisal, reviews were categorized into three groups: “lower” (score 0 to 3); “middle” (score 4 to 7); and “upper” (score 8 to 11). These groups reflect the existence of “major”, “moderate”, and “minor or no methodological limitations” in the included reviews, respectively [14].

### Data extraction and analysis

Two reviewers (SK & GP) independently extracted data from each SR using an electronic form that was developed for the purposes of this study. All extracted data were checked for consistency and any differences were resolved by discussion. Extracted data included general information about the SR (e.g. year of publication, origin, journal, and sources of funding) as well as specific details about the participants, interventions, comparison groups, and outcomes (PICO) of the studies included in the reviews. Citation analyses cross-linking individual SRs with included studies were carried out as a means of identifying the total number of primary studies by study design and type of diabetes, as well as evaluating the degree of overlap between the SRs. Results were analyzed and summarized narratively taking into consideration the methodological quality, scope, and PICO characteristics of each SR.

## Results

**Fig 1:**
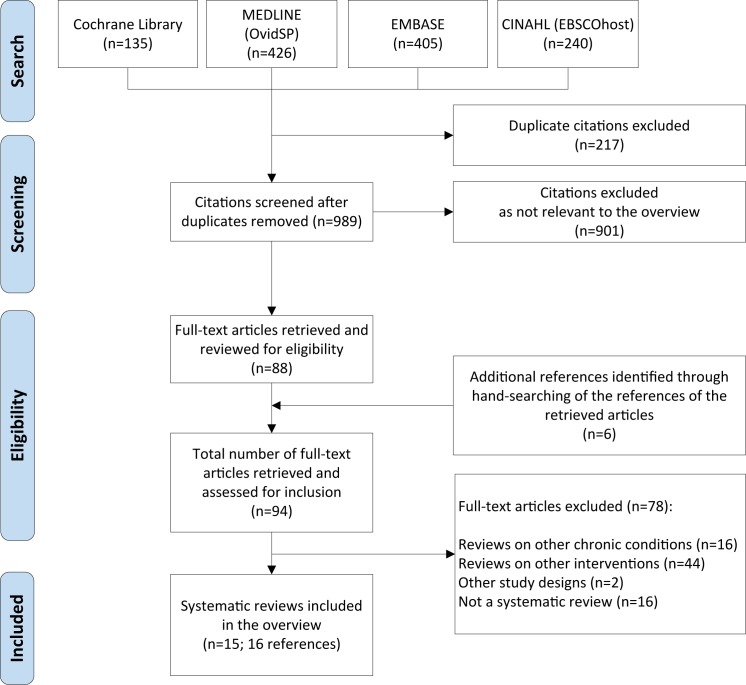
Screen and selection process.

Our initial search yielded 989 citations after removal of 217 duplicates (Fig 1). Based on titles and abstracts, we excluded 901 references that clearly did not meet our inclusion criteria. We then retrieved the full-text of the remaining 88 articles for further examination and manually screened their references to identify any relevant systematic reviews that were not captured by the original search strategy. This process yielded another 6 articles. After close examination, we excluded 78 articles that did not meet our eligibility criteria. The references of the excluded articles along with the primary reasons of exclusion are available in S3 Appendix. Overall, we identified 15 SRs eligible for inclusion [20–34], over 16 references. One Cochrane SR [30] was also published as a journal article [35].

### Description of the included systematic reviews

Reviews included in our sample were published between 2008 and 2014. Most (n = 9) were published from 2012 onward with one third of the reviews being published in 2013 alone. Two of the SRs were Cochrane reviews. Six reviews originated in the United States, five in the United Kingdom, two in China, one in Canada, and one in Iran. Despite some overlap, the individual SRs varied in their scope of inquiry and used different inclusion and exclusion criteria for the selection of eligible studies.

As shown in Table 1, seven SRs included only randomized controlled trials (RCT) [22, 23, 30–34]. The remaining reviews included, in addition to RCTs, non-randomized controlled trials and cohort studies with a pre-post design. Two SRs included only studies that involved patients with T1D [23,25]; two reviews focused explicitly on adult patients with T2D [30,32]; and the remaining 11 included studies that involved patients with type 1, type 2, or both types of diabetes. Of these 11 reviews, only 2 analyzed and reported the effects of mHealth interventions separately for each type of diabetes [20, 29].

**Table 1:** Characteristics of the included systematic reviews.

Author (Year)	Number of included studies (study design) and participants	Interventions (Length of Follow-up)	Control Group	Main Findings	
Baron et al (2012) [20]	20 (12 RCTs, 2 RCOTs, 1 CCT, 5 Pre-Post), 1840 pts (T1D: 507 pts, 7 studies; T2D: 1196 pts, 11 studies; T1D&T2D: 137 pts, 1 study)	Transmission of BG readings and other information (e.g. meal content) to an online server via mobile devices and feedback from a HCP (3–12 months)	Standard care, mHealth without HCP feedback, use of web/personal computer, faxing /phoning BG readings	Findings from the studies are somewhat mixed, but do appear to be more consistently positive for patients with T2D	
Buhi et al (2013) [21]	17^§^ (12 RCTs, 2 RCOTs, 3 Pre-Post); 768 pts (T1D: 206 pts, 5 studies; T2D: 427 pts, 11 studies; T1D&T2D: 137 pts, 1 study)	SMS and/or MMS singularly or combined with other intervention strategies (e.g. Internet, personal digital assistants, phone calls, and patient data monitors) (3–12 months)	No information provided	Of the 17 studies, six reported statistically significant improvements in blood glucose concentrations when SMS was utilized	
Cole-Lewis & Kershaw (2010) [22]	6^§^ (4 RCTs, 2 RCOTs); 333 pts (T1D: 198 pts, 4 studies; T2D: 135 pts, 2 studies)	SMS as the primary mode of intervention delivery. Other components included Internet and email (3–12 months)	Usual care, personal digital assistant, internet-based management, email, paper diary	Significant clinical outcomes noted included decrease in HbA1c levels in adolescents and obese and non‐obese adults with diabetes	
de Jongh et al (2012) [23]	2^§^ (all RCTs); 132 pts (all T1D)	Mobile phone applications (e.g. SMS and MMS) to support self-management of diabetes and offer a way for people to communicate important information to HCP and receive feedback (3–12 months)	Usual care, email reminders	Studies did not demonstrate a significant impact from text messaging on HbA1c: MD -0.15% (95% CI:-0.77, 0.47) and quality of evidence is moderate	
Free et al (2013) [24]	13^§^ (9 RCTs, 3 RCOTs, 1 CCT); 906 pts (T1D: 431 pts, 7 studies; T2D: 338 pts, 5 studies; T1D&T2D: 137 pts, 1 study)	Mobile technology (e.g. PDAs and cell phones) for wireless transmission of BG recordings and delivery of therapeutic advice via SMS	Usual care; paper diary; pedometer; access to website; emails; handheld computer without insulin dose adviser	Mobile technology-based interventions for diabetes control that have statistically significant effects are small and of borderline clinical importance: MD -0.27% (95% CI:-0.48, -0.06)	
Herbert et al (2013) [25]	7 (3 RCTs, 1 RCOT, 1 CCT, 2 Pre-Post); 320 pts (all T1D)	SMS interventions primarily targeting blood glucose monitoring reminders or assessment; other diabetes-related text message topics included use of insulin, nutrition and healthy eating, and physical activity	No information provided	Feasibility was demonstrated across all text message programs, but HbA1c results were mixed. It remains unclear whether or not these programs have an overall long-term influence on daily T1D management	
Holtz and Lauckner (2012) [26]	21 (7 RCTs, 2 RCOTs, 2 CCTs, 6 Pre-Post, 4 feasibility studies; 985 pts (T1D: 697 pts, 13 studies; T2D: 136 pts, 5 studies, T1D&T2D: 146 pts, 2 study; NR: 6 pts, 1 study	Diabetes self-management recommendations via SMS and/or automatic transmission of BG recordings (2 weeks to 12 months)	No information provided	Some positive trends were noted, such as improved self-efficacy and hemoglobin A1c. However, many studies lacked sufficient sample sizes or intervention lengths to determine whether the results might be clinically or statistically significant	
Krishna and Boren (2008) [27]	16 (6 RCTs, 1 RCOT, 9 Pre-Post); 1176 pts (T1D: 251, 7 studies; T2D: 527 pts, 8 studies; Type I & II: 185 pts, 1 study); Note: Included 2 more studies that did not involve patients with diabetes	Cell phones combined with SMS, voice mail, or internet to provide education, personalized advice, reminders to perform diabetes self-management activities, or motivational messages and transfer of blood glucose values from patient to provider	Usual care; conventional support and paper diary; absence of weekly SMS support; conventional insulin therapy; or verbal advice during clinic visit	Providing care and support with cell phones and text message interventions can improve clinically relevant diabetes-related health outcomes by increasing knowledge and self-efficacy	
Krishna et al (2009) [28]	9^§^ (all RCTs); 416 pts (T1D: 195 pts, 4 studies; T2D: 221 pts, 5 studies)	Personalized text messages and voicemail specific to health needs and personal preferences via cell phones; phone reminders, treatment advice, self-care education and personalized goal-specific prompts, motivational tips, and weekly recommendations	Usual care and paper diary; no SMS support or reminder; self-help booklet		All studies but one reported significant improvements in diabetes-related health outcomes. Studies that used weekly recommendations from a nurse to adjust insulin or medication based on information input via SMS showed statistically significant improvements in HbA1c levels
Liang et al (2011) [29]	21 (13 RCTs, 2 RCOTs, 1 CCT, 5 Pre-Post); 1786 pts (T1D: 664 pts, 9 studies; T2D: 800 pts, 10 studies; T1D & T2D: 322 pts, 2 studies)	Most trials used mobile phones with SMS to deliver blood glucose test results and self-management information. Four trials used mobile phone plus Internet. (3–12 months)	No information provided		Studies among T2D patients reported significantly greater reduction in HbA1c than studies among T1D patients (0.8 vs. 0.3%). Smaller trials were more likely to report and publish their results if they found strong effects
Pal et al (2013) [30]	5^§^ (all RCTs); 317 pts (all T2D)	Mobile phone interventions involving reminders, transmission of BG recordings and personalized/tailored text messages about lifestyle, exercise, and medication (3–12 months)	Usual care, face-to-face diabetes education, faxing/phoning BG readings to HCP		Mobile phone interventions involving text-messaging and clinical feedback have a beneficial effect on HbA1c levels for T2D patients: MD -0.5% (95% CI -0.74, -0.26). However, quality of evidence is low
Russell-Minda et al (2009) [31]	9^§^ (all RCTs); 421 pts (T1D: 122 pts, 2 studies; T2D: 259 pts, 6 studies; T1D & T2D: 40 pts, 1 study)	Use of mobile phones with SMS and Internet/Web-based for transmission of BG recordings, reminders, and for diabetes management	No information reported		There was moderately strong evidence from four trials that mobile phones may have helped lower HbA1c levels in patients with type 2 diabetes mellitus
Saffari et al (2014) [32]	10 (9 RCTs, 1CCT); 960 pts (all T2D)	Six studies used SMS for sending and receiving data. In the other studies, only data were received through text-messaging by patients. Four studies used a website along with SMS for sending and receiving data (3–12 months)	No information reported		Diabetes self-management education through text messaging has a considerable effect on HbA1c levels: -0.59% (95% CI: -0.83, -0.35). Effect size was greater among studies that used both SMS and Internet for health education
Sutcliffe et al (2011) [33]	3^§^ (1 RCT, 1 RCOT, 1 Pre-Post); 128 pts (all T1D)	Text messaging or telemedical support via cell phones for the transmission of data and delivery of user feedback, including reminders for diabetes self-management tasks (6 to 8 months)	Usual care alone or with paper-based diary		Communication technologies, including mHealth interventions, may increase the frequency of contact between patient and HCP. Effects on clinical outcomes are unclear
Tao and Or (2013) [34]	13^§^ (12 RCTs; 1 RCOT); 1345 pts (T1D: 447 pts, 5 studies; T2D: 761 pts, 7 studies; T1D & T2D: 137 pts; 1 study)	Automatic upload of clinical data (e.g. HbA1c) to software applications (e.g. websites or other telemonitoring software) installed in the phones and transmission of data to a remote server	Usual care		Mobile phone-based interventions showed statistically significant reductions in HbA1c levels compared to usual care: SMD -0.44% [CI 95%: -0.61, -0.26]

RCT: Randomized Controlled Trial; RCOT: Randomized Crossover Trial; CCT: Controlled Clinical Trial; PTS: Patients; BG: Blood Glucose; HCP: Health Care Providers; SMS: Short Message Service; MMS: Multimedia Message Service

^§^ Number pertains only to mHealth studies focused on diabetes; T1D: type 1 diabetes; T2D: type 2 diabetes.

**Fig 2:**
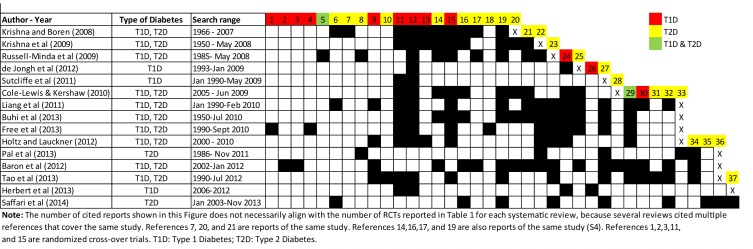
Citation matrix of previously published reports of randomized controlled and cross-over trials included in the systematic reviews (all references are available in S4 Appendix).

**Fig 3:**
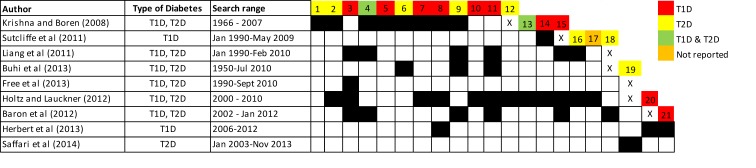
Citation matrix of previously published observational, non-randomized controlled, and uncontrolled trials included in the systematic reviews (all references are available in S4 Appendix).

The number of included studies across the reviews varied significantly (range: 2–21; median: 10 [95% CI: 8 to 15]) due to differences in the inclusion criteria and search strategies. Altogether, the reviews identified 22 unique studies involving patients with T1D (7 RCTs, 5 RCOTs, and 10 with other designs); 26 studies across 31 publications involving patients with T2D (18 RCTs over 23 reports and 8 with other designs); 4 studies involving both T1D and T2D patients (2 RCTs and 2 with other designs); and 1 study that did not specify the type of diabetes of the participants. Figs 2 and 3 present the citation matrices that cross-link with black boxes the individual SRs with all the published reports of the studies included in them. The colored boxes indicate the type of diabetes of the participants included in each of these studies. The full references of the trials are available in S4 Appendix.

Six SRs focused explicitly on the effects of mHealth interventions for patients with diabetes. The remaining reviews had a broader scope of inquiry: 4 SR examined various remote patient monitoring interventions in addition to mHealth (e.g. video and tele-conferencing), and 5 SRs investigated the effects of mHealth on a range of chronic conditions that included diabetes as an identifiable subset. In line with our inclusion criteria, these reviews were included in our sample because they reported the effects of mHealth interventions for diabetes separately from other interventions and/or conditions.

The mHealth interventions included in the SRs were diverse in nature and utilized a wide range of technological innovations, including text messaging (SMS), mobile applications, Bluetooth-enabled glucose meters, as well as secure websites/web-portals that could be accessed through the participants’ mobile device for data entry and patient support. The most prevalent method for sending blood glucose measurements and/or receiving self-management support (e.g. encouragement, education, reminders, and recommendations) involved the use of text messages (SMS) via mobile phones (34 out of 53 studies). Twelve studies involved the use of web-portals that patients accessed through their cell phones to enter diabetes self-care data and receive feedback. In 19 studies, data entry and transmission of glucose data were performed automatically via Bluetooth-enabled devices or other connectivity methods (e.g. infrared transmission or attachment of the glucose device to the mobile phone). In the remaining studies data entry was performed manually. The majority of studies included in the SRs (38 out of 53) encouraged self-monitoring of blood glucose combined with other intervention components, such as support of medication adjustment and reinforcement of lifestyle changes. Half of the studies (26 out of 53) provided educational support to patients via SMS (15 studies) and/or the Internet (11 studies). In 28 of 53 trials patient data were transmitted daily or more often. The duration of follow-up ranged from 3 to 12 months.

Glycemic (HbA1c) control was the primary outcome of interest in all the included reviews. Out of the 15 reviews, only 6 reported other outcomes besides HbA1c. These included changes in body weight [23, 26], participant satisfaction [25, 26], quality of life [27, 33], self-efficacy [26, 28, 33], and costs [26, 33]. Five SRs [23, 24, 29, 30, 32] performed a meta-analysis to calculate the effects of mHealth interventions on HbA1c. The remaining reviews used narrative/qualitative synthesis approaches.

### Methodological quality of the included systematic reviews

As shown in Table 2, the methodological quality of the SRs varied considerably and the majority of them had important limitations. Only 3 reviews were found to be of high quality, achieving a score of 8 or more on the 11-point AMSTAR scale [23, 24, 30]. These reviews adhered to all or most of the methodological criteria, indicating minimal bias in their design and execution. Of the three high quality reviews, two were Cochrane SRs. Cochrane reviews are internationally recognized as the highest standard in evidence-based health care, with rigorous procedures and reporting requirements. Six reviews were of moderate quality, scoring between 4 and 7 AMSTAR points, and six reviews were of low quality scoring less than 4 points. As shown in Table 2, the majority of SRs performed poorly in several domains. All but one SR (n = 14, 93%) failed to assess the sources of support or conflict of interest in the included studies (Q11) and 80% (n = 12) did not provide evidence of a published a priori protocol (Q1). Also, most reviews (n = 12, 80%) did not search the grey literature for eligible studies (e.g. conference proceedings) and restricted their search to English articles only (Q4), which in turn increases the risk for publication and language bias. Only 3 SRs provided a list of excluded studies along with reasons for exclusion of each study (Q5). In more than half of the reviews (n = 8, 53%) there were no details on the number of reviewers involved in, or the process used for, the selection of studies and data extraction, which also raises concerns about the potential subjectivity of decisions at these stages. Finally, one third of the reviews did not appraise the quality of the included trials to identify potential sources of bias (Q7), and approximately half of the included SRs formulated conclusions without taking into consideration the scientific quality of the included trials (Q8). Hence, the possibility that biased studies have inflated the results of these SRs cannot be ruled out. In addition to the methodological issues identified through the AMSTAR instrument, during data extraction and citation analysis we found that three reviews [21, 28, 31], double-counted the results of single trials with duplicate publications and companion papers describing the same study.

**Table 2:** Methodological quality of systematic reviews based on AMSTAR criteria^1^^,^^2^.

Authors (Year)	Q1	Q2	Q3	Q4	Q5	Q6	Q7	Q8	Q9	Q10	Q11	Total
Pal et al (2013) [30]	Y	Y	Y	Y	Y	Y	Y	Y	Y	Y	Y	11
de Jongh (2012) [23]	Y	Y	Y	Y	Y	Y	Y	Y	Y	N	N	9
Free et al (2013) [24]	Y	Y	Y	N	Y	Y	Y	Y	Y	Y	N	9
Liang et al (2011) [29]	N	Y	Y	N	N	Y	Y	Y	Y	Y	N	7
Sutcliffe et al (2011) [33]	N	Y	Y	Y	N	Y	Y	Y	Y	N	N	7
Tao and Or (2013) [34]	N	Y	Y	N	N	Y	Y	Y	Y	Y	N	7
Saffari et al (2014) [32]	N	Y	Y	N	N	Y	Y	N	Y	Y	N	6
Russell-Minda et al (2009) [31]	N	CA	Y	N	N	Y	Y	Y	Y	N	N	5
Cole-Lewis & Kershaw (2010) [22]	N	CA	Y	N	N	Y	Y	N	Y	N	N	4
Baron et al (2012) [20]	N	N	CA	N	N	Y	Y	Y	N	N	N	3
Holtz and Lauckner (2012) [26]	N	CA	Y	N	N	Y	N	N	N	N	N	2
Krishna and Boren (2008) [27]	N	CA	N	N	N	Y	N	N	Y	N	N	2
Buhi et al (2013) [21]	N	CA	CA	N	N	Y	N	N	N	N	N	1
Herbert et al (2013) [25]	N	CA	CA	CA	N	Y	N	N	N	N	N	1
Krishna et al (2009) [28]	N	CA	N	N	N	Y	N	N	N	N	N	1
% of SRs meeting each criterion	20%	47%	67%	20%	20%	100%	67%	53%	67%	33%	7%	μ = 5

^1^Q1: A priori design; Q2: Duplicate study selection and data extraction; Q3: Search comprehensiveness; Q4: Inclusion of grey literature (e.g. non-English articles, and conference proceedings); Q5: Included and excluded studies provided; Q6: Characteristics of the included studies provided; Q7: Scientific quality of the primary studies assessed and documented; Q8: Scientific quality of included studies used appropriately in formulating conclusions; Q9: Appropriateness of methods used to combine studies’ findings; Q10: Likelihood of publication bias was assessed; Q11: Conflict of interest–potential sources of support were clearly acknowledged in both the systematic review and the included studies.

^2^ “Y” (Yes): Criterion met; “N” (No): Criterion not met; CA: Cannot answer; We awarded one point to each item that scored “yes” and summed these to calculate a total score for each review.

### Effects of mHealth interventions on glycosylated hemoglobin (HbA1c)

Of the 15 SRs, four reported the effects of mHealth interventions on HbA1c separately for patients with T1D [20, 23, 25, 29]. Two of the four reviews pooled study results into a meta-analysis and achieved higher methodological quality scores (AMSTAR score > 7) [23, 29]. The review by de Jongh et al [23] investigated the effects of mobile phone messaging interventions utilizing only SMS or MMS to facilitate patient to health care provider communication and self-management of long-term illnesses, including diabetes. Pooled studies (n = 2 RCTs, 132 patients) did not demonstrate a significant impact from text messaging on HbA1c [MD -0.15% (95% CI: -0.77, 0.47], and the overall quality of evidence was ranked as “moderate” due to the small number of included studies and participants. Hence, strong conclusions on the effectiveness of text messaging in supporting self-management of diabetes could not be drawn. The review by Liang et al [29] had a slightly broader scope of inquiry and included studies that combined SMS messages with Internet applications (e.g. web-based applications) for the transmission of self-monitored blood glucose data (daily or more often), reinforcement of lifestyle management including diet and exercise intervention, and delivery of education and medication adjustment. Pooled results from 9 studies (645 patients) showed positive, but clinically and statistically insignificant reductions on HbA1c [0.3% (95% CI: 0.0, -0.5%)], with high statistical heterogeneity among study results in terms of magnitude of effects (I^2^ = 67.5%). The two SRs that synthesized study results narratively and achieved lower methodological quality scores [20, 25], concluded that findings across the included trials were somewhat mixed for T1D patients and that it remained unclear whether text-messaging interventions via cell phones have an overall long-term influence on daily T1D management.

With respect to T2D, out of the 15 SRs only four analyzed and reported results separately [20, 29, 30, 32]. Three reviews performed meta-analysis, while one review synthesized results narratively. The Cochrane review by Pal et al, which achieved the highest AMSTAR score (11), found that mobile phone interventions involving text-messaging and clinical feedback have a beneficial effect on glycemic control [MD of -0.5% (95% CI: -0.74, -0.26), 3 trials, 280 patients]. However, quality of evidence was ranked as “low” by the review authors due to the indirectness of outcome measures and small number of included studies. In a larger meta-analysis that included 10 trials of T2D patients, Liang et al [29] found strong evidence that mobile-phone based interventions involving transmission of BG measurements and clinical feedback, lead to statistically significant improvements in glycemic control [MD -0.8% (95% CI: -1.11, -0.5%). Similar results were also found in a more recent review by Saffari et al [32] (MD 0.59% [95% CI: -0.83 to -0.35] 10 studies, 960 participants]. The authors of this review noted that the effect size was greater among mHealth studies that combine text-messaging with the capabilities of the Internet for the delivery of health education compared to SMS alone (MD -0.85% vs -0.43%). However, statistical heterogeneity was high among study results in both of these SRs [29, 32] (I^2^>65%), suggesting that in future studies the magnitude of intervention effect may vary considerably depending on other study characteristics. In addition, both SRs [29, 32] found evidence of publication bias, with smaller trials being more likely to report and publish their results if they found strong effects. For their part, Baron et al [20] concluded that studies involving T2D patients appear to be more consistently positive compared to T1D.

Of the 9 SRs that examined the effectiveness of mHealth interventions without differentiating between T1D and T2D in the analysis and synthesis of results, 7 reviews reported improvements in HbA1c levels with the intervention [22, 24, 26–28, 31, 34], and two reviews had mixed results [21, 33]. The review by Free et al [24] which achieved the highest AMSTAR score (9) found that mobile technology-based interventions improve glycemic control, but the effects were small and of borderline significance (MD -0.27% (95% CI:-0.48, -0.06), 5 trials, I^2^ = 8.5%).

## Discussion

This umbrella review identified, critically appraised, and synthesized evidence from 15 SRs that evaluated the effectiveness of mHealth interventions, involving the use of mobile devices and applications for remote patient monitoring and delivery of clinical feedback for self-management of diabetes. The reviews included 52 unique studies, most of which were RCTs, published between 1996 and 2014, and evenly distributed between T1D and T2D. To our knowledge, this is the first overview of SRs to pool together in a single source 18 years of mHealth research evidence in the area of diabetes. A major strength of this overview is the use of rigorous methods suggested by the Cochrane Collaboration [17] with the intent to minimize the impact of bias arising from the different sources within and across the included SRs, as well as the overview process itself. More specifically, we carried out a comprehensive search to identify all relevant SRs, performed study selection, data extraction, and quality appraisal in duplicate manner and used a structured, standardized approach that has been developed to help synthesize and rank the evidence across SRs with complex and diverse interventions [14,17]. Nonetheless, the results of this overview are inevitably constrained by the methodological quality and reporting characteristics of the included SRs [36], as well as the quality of evidence within these reviews. Using the AMSTAR instrument, we found that the methodological quality of SRs in this area is suboptimal. Nearly half of the SRs (n = 6; 40%) are characterized by important methodological limitations and risks of bias that impair their internal validity and limit their usefulness for clinical and policy decision-making purposes. In addition to the methodological quality, another key issue that was identified relates to the grouping of T1D and T2D studies in the analysis and synthesis of results. Despite important differences in the demographic characteristics and management approach of T1D and T2D patients, most SRs in our sample (60%, n = 9) mixed the results of the trials and did not differentiate the effects of mHealth interventions between types of diabetes. This is an important limitation that influenced the results of these reviews and made the interpretation of their findings difficult due to the clinical heterogeneity of the included studies. Taken collectively, the findings of our critical appraisal highlight an urgent need to further improve the rigor of future SRs of mHealth interventions in the area of diabetes.

Focusing on the high-quality SRs that offered the most direct evidence, this overview demonstrates that on average, mobile phone-based interventions with clinical feedback improve glycemic control (HbA1c) compared to standard care or other non-mHealth approaches by as much as 0.8% for patients with T2D and 0.3% for patients with T1D, at least in the short-term (≤12 months). The available evidence effectively rules out relatively larger treatment effects (HbA1c >1%). Nonetheless, these findings are consistent with clinically relevant improvements, particularly with respect to patients with T2D [37]. Based on the exploratory subgroup analyses of two reviews [29, 32], mean reductions in HbA1c appear to be more pronounced in interventions that combine text-messaging with Internet functionalities for self-monitoring of blood glucose and delivery of clinical feedback, including health education. Stand-alone text-messaging interventions (single media approach), although effective, have a smaller effect on HbA1c by as much as 0.4%. Also, studies with daily intervention frequency and interactive communication between patients and providers reported greater reductions in HbA1c than those with weekly intervention frequency or unidirectional data collection approaches [29, 32]. However, these findings are exploratory in nature and therefore, should be interpreted with caution. At best, they should be tested as new hypotheses in future trials. Limitations in the quality of evidence such as risk of bias in the clinical trials included in the SRs, statistical heterogeneity between study results in terms of magnitude of effects, and evidence of publication bias, suggest that future research is very likely to have an important impact on our confidence and change these estimates of effect. The presence of publication bias in the available evidence should be further explored in future SRs. The reviews that found evidence of publication bias on the basis of statistical tests and visual inspection of funnel plots did not search for eligible studies in the grey literature [29, 32, 34]. Hence, the possibility that publication bias may have been an artefact of the search limitations of the reviews rather than non-publication of small studies with small or negative effects cannot be excluded.

Patients with T1D represent a unique population with challenging circumstances. Studies including adolescents and young adults with T1D frequently have higher HbA1c levels overall due to hormonal fluctuations, difficulty with increasing demands of diabetes self-care, resistance to parental and health care provider involvement, competing priorities with school or work, and personal life/social stressors [38]. Under these circumstances, mHealth interventions may have limited impact on glycemic control, similar to other traditional behavioral interventions studied in the past over a longer period of time [37]. However, as younger age groups demonstrate higher utilization of mobile devices for most communications, it is compelling for researchers to further investigate the impact of real-time, multi-media approaches to diabetes self-management. Strategies such as gamification, decision aids, and social media among others should systematically be explored in future trials, beyond efforts to simply increasing self-monitoring and transmitting monitoring results [11]. Future studies should also explore the use and impact of diabetes mobile apps that are publicly available through the Apple and Google Play stores. Most trials included in the SRs involved proprietary applications that are not available to the general public. However, as the rates of smartphone ownership and availability of diabetes self-management apps increase [39, 40], it is important to investigate the safety and clinical effectiveness of these apps.

Prior research has shown that when mHealth interventions are designed with a theoretical basis and the content of the intervention is dynamic and adaptive to the needs of each individual, the outcomes are more likely to be successful [22,41]. However, the findings of this review indicate that there is insufficient evidence to determine whether mHealth interventions that are adaptive in nature or based on behavioral change theories are more effective than others. Only few primary studies included in the SRs specified a theoretical basis for the intervention and none of the SRs attempted to explore the impact of theory-based methods and interventions on clinical outcomes. It is not clear whether this is an issue of underreporting in the original studies or lack of theoretical basis due to well-established practice guidelines for managing T1D and T2D [41]. Future empirical studies should improve clarity of reporting with respect to the use of behavioral change theories or models, and other relevant intervention details (e.g. use of adaptive, interactive, or dynamic interventions). Subsequently, SRs should attempt to examine and synthesize evidence on factors related to behavioral change, attitude towards technology and perceived value in order to inform the design of future interventions and optimize their success. In order to leverage mHealth technologies effectively, it is important to understand the mechanisms of behavioral change and factors that affect the adoption of these technologies by patients. The use or lack of theoretical models may be used to better understand and explain variations in the effectiveness of mHealth interventions for diabetes across different age groups (heterogeneity of treatment effects). It is also important to further investigate and build evidence on the optimal type of mHealth interventions (i.e. “modality” of SMS, reminders, education etc.) that would be most effective across different types of patients, with various characteristics. Although mHealth interventions may increase the frequency of contact between patients and health care providers, a more thorough assessment of the behavioral change among providers and the impacts of these interventions on the process of care should also be closely examined.

There is also necessity for further development of research that investigates the impact of mHealth interventions on the utilization of health care services (e.g., emergency room visits and hospitalizations). With the continuous increase in health care costs and focus on quality, several health care systems face the challenge of caring for a constantly growing number of diabetes patients at minimal cost. As a result, a shift of patient care away from health care organizations is necessary to reduce congestion in these settings and to diminish costs. Alike home telemonitoring programs, mHealth represents a promising approach for achieving these objectives. Yet, systematic evaluation of its effects on the consumption of conventional health care services has not been conducted so far to support its wide diffusion. Studies included in the SRs provided little information about the cost of the interventions and did not include cost-effectiveness evaluations, preventing managers and policy makers from confirming their economic viability [20, 22, 29–31]. Importantly, no observations were made in relation to the effects of mHealth on health care providers, their acceptance of this approach, and their concerns about it, which are important issues to consider in future studies [26]. Further, a comparison between the time spent by health care providers delivering individual feedback and tailored advice via email or SMS and the time spent by them otherwise caring for exacerbated cases and complications that could have been minimized by the use of mHealth interventions is worth examining to have a clearer idea of the actual long-term and overall effects of this approach on clinicians’ workloads. The treatment effects found in SRs with mHealth interventions would be important if they could be achieved and sustained across the general population via text messaging and Internet technologies at very low cost, but far from cost-effective if they required significant support from nurses and other HCP and/or additional drugs [30]. When considering the abovementioned issues, future randomized controlled trials should consider adequately-powered samples of patients and be conducted over longer periods of time to be able to draw firm conclusions regarding both the perceived and objective impacts of mHealth interventions on patients with diabetes [26].

## Supporting information

S1 Appendix Search Strategy. (DOCX)

S2 Appendix AMSTAR Operationalization. (DOCX)

S3 Appendix Excluded articles with reasons for exclusion. (DOCX)

S4 Appendix Primary studies included in the systematic reviews. (DOCX)
