# Supplementary material for: Effectiveness of mHealth interventions for patients with diabetes: An overview of systematic reviews
Source: PLoS One. 2017 Mar 1;12(3):e0173160. doi: 10.1371/journal.pone.0173160 (PMC5332111; doi:10.1371/journal.pone.0173160)
Supplement: S1 Appendix — (DOCX) [file pone.0173160.s001.docx]

**Appendix 1: Search Strategy**

**MEDLINE (OvidSP)**

1. exp Diabetes Mellitus/
2. diabet$.tw,ot.
3. (IDDM or NIDDM or MODY or T1DM or T2DM or T1D or T2D).tw,ot.
4. (non insulin$ depend$ or noninsulin$ depend$ or non insulin?depend$ or noninsulin?depend$).tw,ot.
5. (insulin$ depend$ or insulin?depend$).tw.ot
6. ((typ$ 1 or typ$ 2) adj6 diabet$).tw.
7. ((typ$ I or typ$ II) adj6 diabet$).tw.
8. or/1-7
9. exp Diabetes Insipidus/
10. diabet$ insipidus.tw,ot.
11. 9 or 10
12. 8 not 11
13. exp Telecommunications/
14. Telemedicine/
15. Remote Consultation/
16. exp Telemetry/
17. exp Cellular Phone
18. Modems/
19. Telenursing/
20. Monitoring, Physiologic/
21. Monitoring, Ambulatory/
22. Patient Care Planning/
23. Case Management/
24. Home Care Services/
25. Home Care Services, Hospital-Based/
26. Disease Management/
27. (telemetr$ or tele-metr$ or telemed$ or tele-med$ or telehealth$ or tele-health$ or telecare or tele-care or telehome or tele-home or telemonit$ or tele-monit$ or teleconsult$ or tele-consult$ or teleconferenc$ or tele-conferenc$ or telecommunicat$ or tele-communicat$ or telenurs$ or tele-nurs$ or teleservic$ or tele-servic$ or videoconferenc$ or video-conferenc$ or telemanagement or tele-management or telephon$ or phone$ or web-based or web?based or internet-based or internet?based or website or web$site).tw.
28. (remote$ adj3 (consult$ or monitor$)).tw.
29. disease management.tw.
30. 13 or 14 or 15 or 16 or 17 or 18 or 19 or 20 or 21 or 22 or 23 or 24 or 25 or 26 or 27 or 28 or 29
31. 12 and 30
32. ("review" or "review academic" or "review tutorial").pt.
33. (MEDLINE or medlars or embase or pubmed).tw,sh.
34. (scisearch or psychinfo or psycinfo).tw,sh.
35. (psychlit or psyclit).tw,sh.
36. cinahl.tw,sh.
37. ((hand adj2 search$) or (manual$ adj2 search$)).tw,sh.
38. (electronic database$ or bibliographic database$ or computeri?ed database$ or online database$).tw,sh.
39. (pooling or pooled or mantel haenszel).tw,sh.
40. (retraction of publication or retracted publication).pt.
41. (peto or dersimonian or der simonian or fixed effect).tw,sh.
42. 33 or 34 or 35 or 36 or 37 or 38 or 39 or 40 or 41
43. 32 and 42
44. meta-analysis.pt.
45. meta-analysis.sh.
46. (meta-analys$ or meta analys$ or metaanalys$).tw,sh.
47. (systematic$ adj5 review$).tw,sh.
48. (systematic$ adj5 overview$).tw,sh.
49. (quantitativ$ adj5 review$).tw,sh.
50. (quantitativ$ adj5 overview$).tw,sh.
51. (quantitativ$ adj5 synthesis$).tw,sh.
52. (methodologic$ adj5 review$).tw,sh.
53. (methodologic$ adj5 overview$).tw,sh.
54. (integrative research review$ or research integration).tw.
55. 44 or 45 or 46 or 47 or 48 or 49 or 50 or 51 or 52 or 53 or 54
56. 43 or 55
57. 31 and 56

**CINAHL (EBSCOHost)**

1. (MH "Diabetes Mellitus") OR (MH "Diabetes Mellitus, Type 1") OR (MH "Diabetes Mellitus, Type 2") OR (MH "Glucose Metabolism Disorders") OR (MH "Pregnancy in Diabetes") OR (MH "Diabetes Mellitus, Gestational") OR (MH "Prediabetic State")
2. TX diabet*
3. TX (IDDM or NIDDM or MODY or T1DM or T2DM or T1D or T2D)
4. TX (non insulin* depend* or noninsulin* depend* or non insulin?depend* or noninsulin?depend*)
5. TX (insulin* depend* or insulin?depend*)
6. TX ((typ* 1 or typ* 2) N6 diabet*)
7. TX ((typ* I or typ* II) N6 diabet*)
8. Or/1-7
9. (MH "Diabetes Insipidus+")
10. TX (diabet* insipidus)
11. 9 or 10
12. 8 not 11
13. (MH "Monitoring, Physiologic") OR (MH "Blood Glucose Monitoring") OR (MH "Blood Glucose Self-Monitoring") OR (MH "Telemetry") OR (MH "Nursing Assessment")
14. (MH "Patient Care") OR (MH "Case Management") OR (MH "Disease Management") OR (MH "Home Health Care") OR (MH "Ambulatory Care")
15. Or/13-14
16. (MH "Telecommunications") OR (MH "Interactive Voice Response Systems") OR (MH "Teleconferencing") OR (MH "Telecommuting") OR (MH "Telehealth") OR (MH "Telemedicine") OR (MH "Remote Consultation") OR (MH "Telenursing") OR (MH "Telephone") OR (MH "Text Messaging") OR (MH "Videoconferencing") OR (MH "Wireless Communications")
17. TX (telemetr* or tele-metr* or telemed* or tele-med* or telehealth* or tele-health* or telecare or tele-care or telehome or tele-home or telemonit* or tele-monit* or teleconsult* or tele-consult* or teleconferenc* or tele-conferenc* or telecommunicat* or tele-communicat* or telenurs* or tele-nurs* or teleservic* or tele-servic* or videoconferenc* or video-conferenc* or telemanagement or tele-management or telephon* or phone* or web-based or web?based or internet-based or internet?based or website or web*site)
18. TX remote N3 (cosnult* or monitor*)
19. TX disease management
20. Or 16-19
21. TX (MEDLINE)
22. ((MH "Systematic Review")) OR (TX (systematic review))
23. (MH "Meta Analysis")
24. TI (intervention*)
25. Or/21-24
26. 12 AND 15 AND 20 AND 25 (Limiters - Published Date: 19960101-20141131; Exclude MEDLINE records)

**Cochrane Library**

#1 MeSH descriptor: [Diabetes Mellitus] explode all trees

#2 diabet*:ti,ab,kw

#3 (IDDM or NIDDM or MODY or T1DM or T2DM or T1D or T2D):ti,ab,kw

#4 (non insulin* depend* or noninsulin* depend* or non insulin?depend* or noninsulin?depend*):ti,ab,kw

#5 (insulin* depend* or insulin?depend*):ti,ab,kw (Word variations have been searched)

#6 ((typ* 1 or typ* 2) near diabet*):ti,ab,kw

#7 ((typ* I or typ* II) near diabet*):ti,ab,kw

#8 #1 or #2 or #3 or #4 or #5 or #6 or #7

#9 MeSH descriptor: [Diabetes Insipidus] explode all trees

#10 diabet* insipidus

#11 #9 or #10

#12 #8 not #11

#13 MeSH descriptor: [Telecommunications] explode all trees

#14 MeSH descriptor: [Telemedicine] this term only

#15 MeSH descriptor: [Remote Consultation] this term only

#16 MeSH descriptor: [Telemetry] explode all trees

#17 MeSH descriptor: [Cellular Phone] explode all trees

#18 MeSH descriptor: [Modems] this term only

#19 MeSH descriptor: [Telenursing] this term only

#20 MeSH descriptor: [Monitoring, Physiologic] this term only

#21 MeSH descriptor: [Monitoring, Ambulatory] this term only

#22 MeSH descriptor: [Patient Care Planning] this term only

#23 MeSH descriptor: [Case Management] this term only

#24 MeSH descriptor: [Home Care Services] this term only

#25 MeSH descriptor: [Home Care Services, Hospital-Based] this term only

#26 MeSH descriptor: [Disease Management] this term only

#27 telemetr* or tele-metr* or telemed* or tele-med* or telehealth* or tele-health* or telecare or tele-care or telehome or tele-home or telemonit* or tele-monit* or teleconsult* or tele-consult* or teleconferenc* or tele-conferenc* or telecommunicat* or tele-communicat* or telenurs* or tele-nurs* or teleservic* or tele-servic* or videoconferenc* or video-conferenc* or telemanagement or tele-management or telephon* or phone* or web-based or web?based or internet-based or internet?based or website or web*site:ti,ab,kw (Word variations have been searched)

#28 remote near/3 (consult* or monitor*):ti,ab,kw

#29 disease management:ti,ab,kw

#30 #13 or #14 or #15 or #16 or #17 or #18 or #19 or #20 or #21 or #22 or #23 or #24 or #25 or #26 or #27 or #28 or #29

#31 #12 and #30 Publication Year from 1996, in Other Reviews and Technology Assessments

**EMBASE**

1. 'diabetes mellitus'/exp
2. diabet*:ab,ti
3. iddm:ab,ti OR niddm:ab,ti OR mody:ab,ti OR t1dm:ab,ti OR t2dm:ab,ti OR t1d:ab,ti OR t2d:ab,ti
4. or/1-3
5. 'telecommunication'/de
6. 'teleconference'/de
7. 'telehealth'/exp
8. 'telemedicine'/de
9. 'teleconsultation'/de
10. 'telemonitoring'/de
11. ‘telemetry’/exp
12. 'mobile phone'/de
13. 'interactive voice response system'/de
14. 'telephone'/de
15. 'text messaging'/de
16. 'video conferencing'/de
17. 'wireless communication'/de
18. telemetr*:ab,ti OR tele+metr*:ab,ti OR telemed*:ab,ti OR tele+med*:ab,ti OR telehealth*:ab,ti OR tele+health*:ab,ti OR telecare:ab,ti OR tele+care:ab,ti OR telehome:ab,ti OR tele+home:ab,ti OR telemonit*:ab,ti OR tele+monit*:ab,ti OR teleconsult*:ab,ti ORtele+consult*:ab,ti OR teleconferenc*:ab,ti OR tele+conferenc*:ab,ti OR telecommunicat*:ab,ti OR tele+communicat*:ab,ti OR telenurs*:ab,ti OR tele+nurs*:ab,ti OR teleservic*:ab,ti OR tele+servic*:ab,ti OR videoconferenc*:ab,ti OR video+conferenc*:ab,ti ORtelemanagement:ab,ti OR tele+management:ab,ti OR telephon*:ab,ti OR phone*:ab,ti OR web+based:ab,ti OR internet+based:ab,ti OR website:ab,ti OR web*site:ab,ti
19. remote NEAR/3 (consult* OR monitor*)
20. 'disease management':ab,ti
21. 'physiologic monitoring'/de
22. 'ambulatory monitoring'/de
23. 'patient care planning'/de OR 'patient monitoring'/de OR 'case management'/de OR 'home care'/de OR 'home monitoring'/de
24. Or/5-23
25. #4 and #24 (([cochrane review]/lim OR [systematic review]/lim OR [meta analysis]/lim) AND [1996-2014]/py)
