# Supplementary material for: Effectiveness of mHealth interventions for patients with diabetes: An overview of systematic reviews
Source: PLoS One. 2017 Mar 1;12(3):e0173160. doi: 10.1371/journal.pone.0173160 (PMC5332111; doi:10.1371/journal.pone.0173160)
Supplement: S2 Appendix — (DOCX) [file pone.0173160.s002.docx]

| **Appendix 2: AMSTAR Operationalization** | |
| --- | --- |
| **Q1.** | **Was an a priori design provided?**  **Yes**: the authors refer to a published protocol, ethics approval, or pre-determined/a priori published research objectives e.g., SRs for which a research protocol is available (i.e. Cochrane), or SRs developed within specific research programs (HTA, U.S, Preventive Services Task Force, AHQR, NICE etc.)  **No**: the authors do not refer to a protocol, ethics approval or pre-determined/a priori published research objectives and the SR is not developed within specific research programs such as Health Technology Assessment, NICE, AHQR, etc.  **Can’t answer**: the authors refer to a protocol or a set of a priori published research objectives, but the protocol cannot be retrieved or is no longer available. |
| **Q2.** | **Was there duplicate study selection and data extraction?**  **Yes**: at least two people working independently selected studies for inclusion AND extracted the data from the included studies and the method was reported for reaching consensus if disagreements arose.  **No:** the authors stated that both selection and extraction were performed by one person, or that only one of the two processes (i.e. data extraction or study selection) was performed by one person only instead of two (regardless if the second person checked data and/or selection for consistency).  **Can’t answer**: no information about it |
| **Q3.** | **Was a comprehensive literature search performed?**  **Yes:** at least two electronic sources were searched (Cochrane register/Central counts as 2 sources); names of the databases, years searched and keywords and/or search strategy were provided; and the search was supplemented by one of the following strategies: searching of the reference lists of included studies or specialized registers; contacting experts; or searching for grey literature.  **No:** only one database searched or used more than 2 sources but did not supplement searches with any of the above strategies.  **Can’t answer** - partial or no information reported (e.g. databases reported, but keywords or years missing) |
| **Q4.** | **Was status of publication (e.g. grey literature) used as an inclusion criterion?**  **Yes:** the authors explicitly stated that they did not exclude reports based on their language and that they searched for articles irrespective of publication type. If the authors do not explicitly state whether grey literature was used as an inclusion criterion but in the review there exist at least one included report/study that can be classified as non-English article, unpublished, or grey literature (e.g. dissertation, non-peer reviewed conference proceedings), then select Yes.  **No:** authors stated that they excluded studies from the review (or did not search for studies) based on publication status, or language.  **Can’t answer:** no information are provided and no grey literature studies are included in the review |
| **Q5.** | **Was a list of studies (included and excluded provided)?**  **Yes**: a list with the references of the included studies was provided AND a list with the references of the excluded studies (references) was provided either in the article or in a supplementary source (e.g. Appendix, online). The term excluded studies refers to those studies seriously considered by the review authors on the basis of title and/or abstract, but rejected after reading the body of the text.  **No:** only the references of included studies provided; number of excluded studies along with a justification provided but reader can’t link the justification with the exact reference/study that was excluded.  **Can’t answer:** partial information (e.g. all or some of the excluded studies were listed in the article’s references but not in the text to allow the reader identify all of them) |
| **Q6.** | **Were the characteristics of the included studies provided?**  **Yes**: data on participants, interventions and outcomes were provided, and the range of relevant characteristics reported either in a table or as narrative text.  **No:** no information about the characteristics of the included studies provided. For example, review provided information about the interventions but not about the number of participants and the outcomes of interest of the study.  **Can’t answer:** partial information (e.g. only year of publication and intervention reported, or only some of the included studies described) |
| **Q7.** | **Was the scientific quality of the included studies assessed and reported?**  **Yes:** predetermined methods of assessing quality were reported i.e. a risk of bias or methodological quality assessment instrument/tool was used to critically appraise each study against the instrument’s criteria with some kind of result reported for each study.  **No:** no quality assessment performed on the actual features of the individual studies (e.g. randomization, concealment of allocation, blinding of assessors, attrition, and/or other study design and implementation characteristics).  **Can’t answer:** the authors stated that a quality assessment was done, but did not describe how it was performed (e.g. what instruments or criteria were used) and/or do not present the results of the assessment. |
| **Q8.** | **Was the scientific quality of the included studies used appropriately in formulating conclusions?**  **Yes:** the quality (and limitations) of included studies was considered in the analysis (e.g. use of the GRADE system to rate the quality of evidence for each outcome) and/or the conclusions of the review (i.e. in making inferences about the effectiveness of mHealth). For example, authors might say “the results should be interpreted with caution due to the poor quality of the included studies”.  **No**: quality assessment was not performed or was but the results were not considered throughout the analysis of the findings and/or at the end in formulating conclusions.  **Can’t answer**: impact of quality of studies on results unclear or not used for conclusions. |
| **Q9.** | **Were the methods used to combine the findings of studies appropriate?**  **Yes:** In SRs that pooled the results using meta-analysis, if statistical heterogeneity was assessed by means of a formal test (e.g., Chi-squared and/or I²) and the results of these tests - along with other study aspects such as the clinical heterogeneity between the interventions - were used to inform the decision of the statistical model used (i.e. random or fixed). If statistical heterogeneity was present, (given the nature of mHealth interventions) a random effects model was used and/or the appropriateness of combining data was considered by the review authors. Yes, also, if in SRs that did not conduct meta-analysis, the authors made a statement regarding the inappropriateness of pooling data (e.g. highlighted issues about heterogeneity/variability between the studies) and thus, a qualitative synthesis was performed appropriately. That is, the authors summarized and synthesized the available evidence narratively according to a defined analysis plan and/or using appropriate qualitative methods and techniques (e.g. construction of common rubrics, content analysis, tabulation, groupings and clustering).  **No:** In SRs that pooled the results using meta-analysis, heterogeneity was present, but not discussed, fixed-effect model was used by default, and/or meta-analytic methods were used inappropriately (double counting of studies occurred, count data were treated as dichotomous, etc.). **Note**: if there is no heterogeneity present (e.g. I²=0% or chi-square is non-significant, P is greater than 0.10), and review used fixed-effect model, score Yes because both fixed and random effects models yield the same results in this case. No, also, in SRs in which the authors did not attempt to combine findings into a meta-analysis and did not provide a statement regarding heterogeneity or the inappropriateness of combining findings.  **Can’t answer:** heterogeneity test result not reported or model (random vs. fixed) used to combine studies not specified. |
| **Q10.** | **Was the likelihood of publication bias assessed?**  **Yes:** publication bias was explicitly considered and assessed. Funnel plots or other methods used (e.g. egger regression tests). (Note: if funnel plots are not presented as figures, but authors explicitly state that a publication bias assessment was performed and an interpretation of that test is provided, then score Yes.)  **No:**  In SRs that pooled the results using meta-analysis, publication bias was not assessed or no information about it was provided.  **Can’t answer:** mentioned or discussed it vaguely only in conclusions.  **Not applicable:** SR was narrative/qualitative not a meta-analysis |
| **Q11.** | **Was the conflict of interest stated?**  **Yes:** conflict of interest and sources of support were clearly acknowledged in both the systematic review AND the included studies.  **No:** conflict of interest and sources of funding were reported for the systematic review but not for the included primary studies or vice versa. |
