# Supplementary material for: Effectiveness of mHealth interventions for patients with diabetes: An overview of systematic reviews
Source: PLoS One. 2017 Mar 1;12(3):e0173160. doi: 10.1371/journal.pone.0173160 (PMC5332111; doi:10.1371/journal.pone.0173160)
Supplement: S3 Appendix — (DOCX) [file pone.0173160.s003.docx]

**Appendix 3: Excluded articles with reasons for exclusion**

| **Number** | **Reference** | **Reasons for exclusion** |
| --- | --- | --- |
|  | Angeles RN, Howard MI, Dolovich L. The effectiveness of web-based tools for improving blood glucose control in patients with diabetes Mellitus: A meta-analysis. Canadian Journal of Diabetes. 2011;35:344-52. | Contra to protocol: included studies of non-mHealth interventions |
|  | Anglada-Martinez H, Riu-Viladoms G, Martin-Conde M, Rovira-Illamola M, Sotoca-Momblona JM, Codina-Jane C. Does mHealth increase adherence to medication? Results of a systematic review. International Journal of Clinical Practice. 2015;69(1):9-32. | Contra to protocol: included studies did not involve physiological monitoring and transmission of HbA1c. Focused on medication adherence only |
|  | Azar M, Gabbay R. Web-based management of diabetes through glucose uploads: has the time come for telemedicine? Diabetes Res Clin Pract 2009;83(1):9-17. | Contra to protocol: not a systematic review |
|  | Balas EA, Krishna S, Kretschmer RA, Cheek TR, Lobach DF, Boren SA. Computerized knowledge management in diabetes care. Med Care. 2004;42:610-21. | Contra to protocol: included studies of non-mHealth interventions |
|  | Barlow J, Singh D, Bayer S, Curry R. A systematic review of the benefits of home telecare for frail elderly people and those with long-term conditions. J Telemed Telecare. 2007;13:172-9. | Contra to protocol: included studies involving patients with other chronic diseases. Diabetes results not reported separately |
|  | Bass AM, Farhangian ME, Feldman S.R. Internet-based adherence interventions for treatment of chronic disorders in adolescents. Adolescent Health, Medicine and Therapeutics. 2015;6: 91-99. | Contra to protocol: included studies involving patients with other chronic diseases. Diabetes results not reported separately |
|  | Beatty L, Lambert S. A systematic review of internet-based self-help therapeutic interventions to improve distress and disease-control among adults with chronic health conditions. Clin Psychol Rev. 2013;33:609-22. | Contra to protocol: included studies of non-mHealth interventions as well as studies that are self-guided and do not involve clinical feedback |
|  | Bensink M, Hailey D, Wootton R. A systematic review of successes and failures in home telehealth: preliminary results... 6th International Conference on Successes and Failures in Telehealth. J Telemed Telecare. 2006;12:S3:8-16. | Contra to protocol: included studies with other chronic diseases. Results for each diabetes outcome of interest (e.g. HbA1c, satisfaction, quality of life) were not reported separately |
|  | Biem HJ, Turnell RW, D'Arcy C. Computer telephony: automated calls for medical care. Clin Invest Med 2003;26(5):259-68. | Contra to protocol: not specific to diabetes care; included studies that focused on other chronic conditions (e.g. hypertension and chronic lung diseases) |
|  | Blake H. Mobile phone technology in chronic disease management. Nurs Stand. 2008;23(12):43-6. | Contra to protocol: not a systematic review |
|  | Botsis T, Hartvigsen G. Current status and future perspectives in telecare for elderly people suffering from chronic diseases. J Telemed Telecare. 2008;14:195-203. | Contra to protocol: not a systematic review |
|  | Cassimatis, Kavanagh, David J. Effects of type 2 diabetes behavioural telehealth interventions on glycaemic control and adherence: a systematic review. Journal of Telemedicine and Telecare. 2012;18:447-50. | Contra to protocol: included studies of non-mHealth interventions |
|  | Connelly J, Kirk A, Masthoff J, Macrury S. The use of technology to promote physical activity in Type 2 diabetes management: A systematic review. Diabet Med. 2013;30:1420-32. | Contra to protocol: included studies of non-mHealth interventions as well as studies that did not involve clinical feedback |
|  | Costa BM, Fitzgerald KJ, Jones KM, Dunning A. Effectiveness of IT-based diabetes management interventions: a review of the literature. BMC Fam Pract. 2009;10:72. | Contra to protocol: not a systematic review |
|  | Cotter AP, Durant N, Agne AA, Cherrington AL. Internet interventions to support lifestyle modification for diabetes management: A systematic review of the evidence. Journal of Diabetes and its Complications. 2014;28:243-51. | Contra to protocol: included studies of non-mHealth interventions as well as studies that did not involve clinical feedback |
|  | Dalton JE. Web-based care for adults with type 2 diabetes. Canadian Journal of Dietetic Practice and Research. 2008;69:185-91. | Contra to protocol: not a systematic review |
|  | de Jong CC, Ros WJ, Schrijvers G. The effects on health behavior and health outcomes of Internet-based asynchronous communication between health providers and patients with a chronic condition: a systematic review. J Med Internet Res. 2014;16:e19. | Contra to protocol: included studies involving patients with other chronic diseases. Diabetes results not reported separately |
|  | Dellifraine JL, Dansky KH. Home-based telehealth: a review and meta-analysis. Journal of telemedicine and telecare. 2008;14:62-6. | Contra to protocol: included studies of non-mHealth interventions |
|  | Dennis SM, Harris M, Lloyd J, Powell D, Faruqi N, Zwar N. Do people with existing chronic conditions benefit from telephone coaching? A rapid review. Aust Health Rev. 2013;37:381-8. | Contra to protocol: not a systematic review |
|  | El-Gayar O, Timsina P, Nawar N, Eid W. Mobile applications for diabetes self-management: status and potential. J Diabetes Sci Technol. 2013;7:247-62. | Contra to protocol: included study designs that are out of the scope of this overview of systematic reviews (e.g. case studies) as well as mobile health apps from the Apple store |
|  | El-Gayar O, Timsina P, Nawar N, Eid W. A systematic review of IT for diabetes self-management: are we there yet? Int J Med Inf. 2013;82:637-52. | Contra to protocol: included studies of non-mHealth interventions as well as studies that did not involve clinical feedback |
|  | Elissen AM, Steuten LM, Lemmens LC, Drewes HW, Lemmens KM, Meeuwissen JA, et al. Meta-analysis of the effectiveness of chronic care management for diabetes: investigating heterogeneity in outcomes. J Eval Clin Pract. 2013;19:753-62. | Contra to protocol: included studies of non-mHealth interventions |
|  | Farmer A, Gibson OJ, Tarassenko L, Neil A. A systematic review of telemedicine interventions to support blood glucose self-monitoring in diabetes. Diabet Med. 2005;22:1372-8. | Contra to protocol: included studies of non-mHealth interventions |
|  | Farmer AJ, McSharry J, Rowbotham S, McGowan L, Ricci-Cabello I, French DP. Effects of interventions promoting monitoring of medication use and brief messaging on medication adherence for people with Type 2 diabetes: A systematic review of randomized trials. Diabet. Med. 2016;33: 565–579 | Contra to protocol: included studies did not involve physiological monitoring and transmission of HbA1c. Focused on medication adherence only |
|  | Ferguson S, Swan M, Smaldone A. Does diabetes self-management education in conjunction with primary care improve glycemic control in Hispanic patients? A systematic review and meta-analysis. The Diabetes educator. 2015;41(4):472-84. | Contra to protocol: did not focus on mHealth interventions |
|  | Fitzner K, Moss G. Telehealth--an effective delivery method for diabetes self-management education? Popul Health Manag. 2013;16:169-77. | Contra to protocol: not a systematic review |
|  | Fjeldsoe BS, Marshall AL, Miller YD. Behavior change interventions delivered by mobile telephone short-message service. Am J Prev Med. 2009;36:165-73. | Contra to protocol: included studies involving patients with other chronic diseases. Diabetes results not reported separately |
|  | Flodgren G, Rachas A, Farmer AJ, Inzitari M, Shepperd S. Interactive telemedicine: effects on professional practice and health care outcomes. The Cochrane database of systematic reviews. 2015;9:CD002098. | Contra to protocol: included studies of non-mHealth interventions |
|  | Garabedian LF, Ross-Degnan D, Wharam JF. Mobile Phone and Smartphone Technologies for Diabetes Care and Self-Management. Current Diabetes Reports. 2015;15(12):109 | Contra to protocol: included interventions that did not involve clinical feedback |
|  | Garcia-Lizana F, Sarria-Santamera A. New technologies for chronic disease management and control: a systematic review. J Telemed Telecare. 2007;13:62-8. | Contra to protocol: included studies involving patients with other chronic diseases. Diabetes results not reported separately |
|  | Gray PA, Drayton-Brooks S, Williamson KM. Diabetes: follow-up support for patients with uncontrolled diabetes. Nurse Pract. 2013;38:49-53. | Contra to protocol: not a systematic review. |
|  | Graziano JA, Gross CR. The effects of isolated telephone interventions on glycemic control in type 2 diabetes: a literature review. ANS Adv Nurs Sci. 2009;32:E28-e41. | Contra to protocol: did not focus on mHealth interventions |
|  | Greenwood DA, Young HM, Quinn CC. Telehealth remote monitoring systematic review: Structured self-monitoring of blood glucose and impact on A1C. Journal of Diabetes Science and Technology. 2014;8:378-89. | Contra to protocol: included studies of non-mHealth interventions. Results were not analyzed separately for the mHealth interventions |
|  | Greenwood, D. Telehealth remote patient monitoring intervention for people with type 2 diabetes." Communicating Nursing Research. 2013;46: 715-715. | Contra to protocol: not a systematic review |
|  | Hamine S, Gerth-Guyette E, Faulx D, Green BB, Ginsburg AS. Impact of mHealth chronic disease management on treatment adherence and patient outcomes: a systematic review. Journal of medical Internet research. 2015;17(2):e52. | Contra to protocol: included studies involving patients with other chronic diseases. Diabetes results not reported separately |
|  | Hatherly K, Overland J, Smith L, Taylor S, Johnston C. Providing optimal service delivery for children and adolescents with type 1 diabetes: A systematic review. Practical Diabetes International. 2009;26:154-9. | Contra to protocol: included studies of non-mHealth interventions as well as studies without clinical feedback |
|  | Hayes, Inc. The use of Information Communication Technology (ICT) to improve treatment adherence in patients with diabetes. Health Technology Assessment Database. 2014. | Contra to protocol: included non-mHealth intervention studies |
|  | Health Quality Ontario. Home telemonitoring for type 2 diabetes: an evidence-based analysis. Ont Health Technol Assess Ser. 2009;9:1-38. | Contra to protocol: included non-mHealth intervention studies. Results were not analyzed separately for the mHealth interventions |
|  | Hersh WR, Helfand M, Wallace J, Kraemer D, Patterson P, Shapiro S, et al. Clinical outcomes resulting from telemedicine interventions: a systematic review. BMC Med Inf Decis Mak. 2001;1:5. | Contra to protocol: included studies that involve gestational diabetes patients |
|  | Huang Z, Tao H, Meng Q, Jing L. Effects of telecare intervention on glycemic control in type 2 diabetes: A systematic review and meta-analysis of randomized controlled trials. European Journal of Endocrinology. 2015;172(3):R93-R101. | Contra to protocol: included non-mHealth intervention studies. Results were not analyzed separately for the mHealth interventions |
|  | Hutchison AJ, Breckon JD. A review of telephone coaching services for people with long-term conditions (Provisional abstract). Journal of Telemedicine and Telecare. 2011;17:451-8. | Contra to protocol: not a systematic review |
|  | Jaana M, Pare G. Home telemonitoring of patients with diabetes: a systematic assessment of observed effects (Structured abstract). J Eval Clin Pract. 2007;13:242-53. | Contra to protocol: did not focus on mHealth interventions |
|  | Jackson CL, Bolen S, Brancati FL, Batts-Turner ML, Gary TL. A systematic review of interactive computer-assisted technology in diabetes care: Interactive information technology in diabetes care. J Gen Intern Med. 2006;21:105-10. | Contra to protocol: did not focus on mHealth interventions |
|  | Joe J, Demiris G. Older adults and mobile phones for health: a review. J Biomed Inform. 2013;46:947-54. | Contra to protocol: not a systematic review |
|  | Kuijpers W, Groen WG, Aaronson NK, van H, W H. A systematic review of web-based interventions for patient empowerment and physical activity in chronic diseases: relevance for cancer survivors. J Med Internet Res. 2013;15:e37. | Contra to protocol: included studies involving patients with other chronic diseases |
|  | Lepard MG, Joseph AL, Agne AA, Cherrington AL. Diabetes self-management interventions for adults with type 2 diabetes living in rural areas: a systematic literature review. Current diabetes reports. 2015;15(6):608. | Contra to protocol: included studies in which patients traveled to their primary care clinic for videoconferences. |
|  | Majeed-Ariss R, Baildam E, Campbell M, Chieng A, Fallon D, Hall A, et al. Apps and Adolescents: A Systematic Review of Adolescents' Use of Mobile Phone and Tablet Apps That Support Personal Management of Their Chronic or Long-Term Physical Conditions. Journal of medical Internet research. 2015;17(12):e287. | Contra to protocol: included studies involving patients with other chronic diseases. Results were not analyzed separately for diabetes |
|  | Marcolino MS, Maia JX, Alkmim MBM, Boersma E, Ribeiro A. Telemedicine application in the care of diabetes patients: Systematic review and meta-analysis. Diabetes Technology and Therapeutics. 2015;17:S55-S6. | Contra to protocol: included non-mHealth intervention studies. Results were not analyzed separately for the mHealth interventions |
|  | Mignerat M, Lapointe L, Vedel I. Using telecare for diabetic patients: A mixed systematic review. Health Policy and Technology. 2014;3:90-112. | Contra to protocol: included qualitative and mixed-method studies (e.g. case studies, surveys, and descriptive studies), as well as interventions that did not involve clinical feedback (e.g. social media/forums) |
|  | Montani S, Bellazzi R, Quaglini S, d'Annunzio G. Meta-analysis of the effect of the use of computer-based systems on the metabolic control of patients with diabetes mellitus. Diabetes Technol Ther. 2001;3:347-56. | Contra to protocol: included studies that involved other than mHealth interventions (e.g. decision support, stand-alone education and computerized clinical guidelines interventions) |
|  | Montori VM, Helgemoe PK, Guyatt GH, Dean DS, Leung TW, Smith SA, et al. Telecare for Patients with Type 1 Diabetes and Inadequate Glycemic Control: A randomized controlled trial and meta-analysis. Diabetes Care. 2004;27:1088-94. | Contra to protocol: did not include mHealth interventions |
|  | Moy F, Ming, Ray A, Buckley B. Techniques of monitoring blood glucose during pregnancy for women with pre-existing diabetes. Cochrane Database Syst Rev. 2014. doi: 10.1002/14651858.CD009613.pub2. | Contra to protocol: did not include mHealth interventions |
|  | Mulvaney SA, Ritterband LM, Bosslet L. Mobile intervention design in diabetes: review and recommendations. Curr Diab Rep. 2011;11:486-93. | Contra to protocol: not a systematic review |
|  | Mushcab H, Kernohan WG, Wallace J, Martin S. Web-Based Remote Monitoring Systems for Self-Managing Type 2 Diabetes: A Systematic Review. Diabetes technology & therapeutics. 2015;17(7):498-509. | Contra to protocol: included studies of non-mHealth interventions. Results were not analyzed separately for the mHealth interventions |
|  | Paré G, Jaana M, Sicotte C. Systematic review of home telemonitoring for chronic diseases: the evidence base. J Am Med Inform Assoc. 2007;14:269-77. | Contra to protocol: did not include mHealth interventions |
|  | Paré G, Moqadem K, Pineau G, St-Hilaire C. Systematic review of the effects of home telemonitoring in the context of diabetes, pulmonary diseases and cardiovascular diseases. ETMIS 2009, 5 (3). | Contra to protocol: did not include mHealth interventions |
|  | Paré G, Moqadem K, Pineau G, St-Hilaire C. Clinical effects of home telemonitoring in the context of diabetes, asthma, heart failure and hypertension: a systematic review. J Med Internet Res. 2010;12:e21. | Contra to protocol: did not include mHealth interventions |
|  | Paré G, Moqadem K, Pineau G, St-Hilaire C. Impacts of telemonitoring in the context of diabetes. Medecine des Maladies Metaboliques. 2010;4:257-62. | Contra to protocol: did not include mHealth interventions |
|  | Pereira K, Phillips B, Johnson C, Vorderstrasse A. Internet delivered diabetes self-management education: a review. Diabetes technology & therapeutics. 2015;17(1):55-63. | Contra to protocol: did not include mHealth interventions |
|  | Peterson A. Improving type 1 diabetes management with mobile tools: A systematic review. Journal of Diabetes Science and Technology. 2014;8:859-64. | Contra to protocol: included interventions that did not involve transmission of data from the patient to the provider or clinical feedback |
|  | Polisena J, Coyle D, Coyle K, McGill S. Home telehealth for chronic disease management: A systematic review and an analysis of economic evaluations. Int J Technol Assess Health Care. 2009;25:339-49. | Contra to protocol: included studies involving patients with other chronic diseases. |
|  | Polisena J, Tran K, Cimon K, Hutton B, McGill S, Palmer K. Home telehealth for diabetes management: A systematic review and meta-analysis. Diabetes, Obesity and Metabolism. 2009;11:913-30. | Contra to protocol: did not include mHealth interventions |
|  | Ramadas A, Quek KF, Chan CKY, Oldenburg B. Web-based interventions for the management of type 2 diabetes mellitus: A systematic review of recent evidence. Int J Med Inf. 2011;80:389-405. | Contra to protocol: did not include mHealth interventions |
|  | Riazi H, Larijani B, Langarizadeh M, Shahmoradi L. Managing diabetes mellitus using information technology: A systematic review. Journal of Diabetes and Metabolic Disorders. 2015;14(1). | Contra to protocol: included studies of non-mHealth interventions (e.g. clinical decision support systems and electronic medical records) |
|  | Samoocha D, Bruinvels DJ, Elbers NA, Anema JR, van der B, A J. Effectiveness of web-based interventions on patient empowerment: a systematic review and meta-analysis. J Med Internet Res. 2010;12:e23. | Contra to protocol: included studies involving patients with other chronic diseases. |
|  | Schoenthaler A, Cuffee YL. A systematic review of interventions to improve adherence to diabetes medications within the patient-practitioner interaction. Journal of Clinical Outcomes Management. 2013;20:494-506. | Contra to protocol: did not focus on mHealth interventions |
|  | Shulman RM, O'Gorman CS, Palmert MR. The impact of telemedicine interventions involving routine transmission of blood glucose data with clinician feedback on metabolic control in youth with type 1 diabetes: a systematic review and meta-analysis. Int J Pediatr Endocrinol. 2010;2010:536957. | Contra to protocol: did not focus on mHealth interventions |
|  | Siriwardena LS, Wickramasinghe WA, Perera KL, Marasinghe RB, Katulanda P, Hewapathirana R. A review of telemedicine interventions in diabetes care. J Telemed Telecare. 2012;18:164-8. | Contra to protocol: not a systematic review |
|  | Suksomboon N, Poolsup N, Nge YL. Impact of phone call intervention on glycemic control in diabetes patients: a systematic review and meta-analysis of randomized, controlled trials. PLoS ONE. 2014;9:e89207. | Contra to protocol: did not focus on mHealth interventions |
|  | Tatara N, Årsand E, Nilsen H, et al. A Review of Mobile Terminal Based Applications for Self-Management of Patients with Diabetes. 2009:166-75. | Contra to protocol: not a systematic review |
|  | Tildesley HD, Po MD, Ross SA. Internet Blood Glucose Monitoring Systems Provide Lasting Glycemic Benefit in Type 1 and 2 Diabetes: A Systematic Review. Medical Clinics of North America. 2015;99(1):17-33. | Contra to protocol: included non-mHealth intervention studies. Results were not analyzed separately for the mHealth interventions |
|  | van den Berg, Schumann M, Kraft K, Hoffmann W. Telemedicine and telecare for older patients-A systematic review. Maturitas. 2012;2:94-114. | Contra to protocol: included studies involving patients with other chronic diseases. Results pertaining to patients with diabetes were not reported separately by outcome of interest |
|  | van Vugt M, de Wit M, Cleijne WH, Snoek FJ. Use of behavioral change techniques in web-based self-management programs for type 2 diabetes patients: systematic review. J Med Internet Res. 2013;15(12):e279. | Contra to protocol: did not focus on mHealth interventions |
|  | Verhoeven F, van G-P, Dijkstra K, Nijland N, Seydel E, Steehouder M. The contribution of teleconsultation and videoconferencing to diabetes care: a systematic literature review. J Med Internet Res. 2007;9:e37. | Contra to protocol: included studies that involved gestational diabetes patients; study designs outside the scope of this overview (e.g. expert opinion); and telemedicine interventions |
|  | Verhoeven F, Tanja-Dijkstra K, Nijland N, Eysenbach G, Gemert-Pijnen L. Asynchronous and synchronous teleconsultation for diabetes care: a systematic literature review (Structured abstract). Journal of Diabetes Science and Technology. 2010;4:666-84. | Contra to protocol: included studies that involved gestational diabetes patients; study designs outside the scope of this overview (e.g. expert opinion); and telemedicine interventions |
|  | Wootton R. Twenty years of telemedicine in chronic disease management--an evidence synthesis. J Telemed Telecare. 2012;18:211-20. | Contra to protocol: not a systematic review |
|  | Wu L, Forbes A, Griffiths P, Milligan P, While A. Telephone follow-up to improve glycaemic control in patients with Type 2 diabetes: Systematic review and meta-analysis of controlled trials. Diabet Med. 2010;27:1217-25. | Contra to protocol: did not focus on mHealth interventions |
|  | Zhai YK, Zhu WJ, Cai YL, Sun DX, Zhao J. Clinical- and cost-effectiveness of telemedicine in type 2 diabetes mellitus: A systematic review and meta-analysis. Medicine (United States). 2014;93(28):e312. | Contra to protocol: included non-mHealth intervention studies. Results were not analyzed separately for the mHealth interventions |
