# Supplementary material for: Effectiveness of mHealth interventions for patients with diabetes: An overview of systematic reviews
Source: PLoS One. 2017 Mar 1;12(3):e0173160. doi: 10.1371/journal.pone.0173160 (PMC5332111; doi:10.1371/journal.pone.0173160)
Supplement: S4 Appendix — (DOCX) [file pone.0173160.s004.docx]

**Appendix 4: Primary studies included in the systematic reviews**

**Randomized Controlled and Cross-Over Trials**

1. Holman RR, Smale AD, Pemberton E, Riefflin A, Nealon JL. Randomized controlled pilot trial of a hand-held patient-oriented, insulin regimen optimizer. Med Informatics. 1996;21(4):317–26.
2. Tsang MW, Mok M, Kam G, Jung M, Tang A, Chan U, et al. Improvement in diabetes control with a monitoring system based on a hand-held, touch-screen electronic diary. J Telemed Telecare. 2001;7(1):47–50.
3. Gómez EJ, Hernando ME, García A, Del Pozo F, Cermeño J, Corcoy R, et al. Telemedicine as a tool for intensive management of diabetes: the DIABTel experience. Comput Methods Programs Biomed. 2002;69(2):163–77.
4. Schrezenmeir J, Dirting K, Papazov P. Controlled multicenter study on the effect of computer assistance in intensive insulin therapy of type 1 diabetics. Comput Methods Programs Biomed. 2002;69(2):97–114.
5. Kumar VS, Wentzell KJ, Mikkelsen T, Pentland A, Laffel LM. The DAILY (Daily Automated Intensive Log for Youth) Trial: A Wireless, Portable System to Improve Adherence and Glycemic Control in Youth with Diabetes. Diabetes Technol Ther. 2004;6(4):445–53.
6. Liu C-T, Yeh Y-T, Lee T-I, Li Y-C. Observations on online services for diabetes management. Diabetes Care. 2005;28(11):2807–8.
7. Kim H-S. [Effects of Web-based diabetic education in obese diabetic patients]. Taehan Kanho Hakhoe Chi. 2005;35(5):924–30.
8. Leu MG, Norris TE, Hummel J, Isaac M, Brogan MW. A Randomized, Controlled Trial of an Automated Wireless Messaging System for Diabetes. Diabetes Technol Ther. 2005;7(5):710–8.
9. Farmer AJ, Gibson OJ, Dudley C, Bryden K, Hayton PM, Tarassenko L, et al. A randomized controlled trial of the effect of real-time telemedicine support on glycemic control in young adults with type 1 diabetes (ISRCTN 46889446). Diabetes Care. 2005;28(11):2697–702.
10. Lee T-I, Yeh Y-T, Liu C-T, Chen P-L. Development and evaluation of a patient-oriented education system for diabetes management. Int J Med Inform. 2007;76(9):655–63.
11. Rami B, Popow C, Horn W, Waldhoer T, Schober E. Telemedical support to improve glycemic control in adolescents with type 1 diabetes mellitus. Eur J Pediatr. 2006;165(10):701–5.
12. Franklin VL, Waller A, Pagliari C, Greene SA. A randomized controlled trial of Sweet Talk, a text-messaging system to support young people with diabetes. Diabet Med. 2006;23(12):1332–8.
13. Tasker APB, Gibson L, Franklin V, Gregor P, Greene S. What is the frequency of symptomatic mild hypoglycemia in type 1 diabetes in the young?: Assessment by novel mobile phone technology and computer-based interviewing. Pediatr Diabetes. 2007;8(1):15–20.
14. Kim H-S, Jeong H-S. A nurse short message service by cellular phone in type-2 diabetic patients for six months. J Clin Nurs. 2007;16(6):1082–7.
15. Benhamou P-Y, Melki V, Boizel R, Perreal F, Quesada J-L, Bessieres-Lacombe S, et al. One-year efficacy and safety of Web-based follow-up using cellular phone in type 1 diabetic patients under insulin pump therapy: the PumpNet study. Diabetes Metab. 2007;33(3):220–6.
16. Kim H-S. A randomized controlled trial of a nurse short-message service by cellular phone for people with diabetes. Int J Nurs Stud. 2007;44(5):687–92.
17. Hee-Sung K. Impact of web-based nurse’s education on glycosylated haemoglobin in type 2 diabetic patients. J Clin Nurs. 2007;16(7):1361–6.
18. Kim C, Kim H, Nam J, Cho M, Park J, Kang E, et al. Internet diabetic patient management using a short messaging service automatically produced by a knowledge matrix system. Diabetes Care. 2007;30(11):2857–8.
19. Yoon K-H, Kim H-S. A short message service by cellular phone in type 2 diabetic patients for 12 months. Diabetes Res Clin Pract. 2008;79(2):256–61.
20. Kim H-S, Song M-S. Technological intervention for obese patients with type 2 diabetes. Appl Nurs Res. 2008;21(2):84–9.
21. Kim S-I, Kim H-S. Effectiveness of mobile and internet intervention in patients with obese type 2 diabetes. Int J Med Inform. 2008;77(6):399–404.
22. Quinn CC, Clough SS, Minor JM, Lender D, Okafor MC, Gruber-Baldini A. WellDoc mobile diabetes management randomized controlled trial: change in clinical and behavioral outcomes and patient and physician satisfaction. Diabetes Technol Ther. 2008;10(3):160–8.
23. Faridi Z, Liberti L, Shuval K, Northrup V, Ali A, Katz DL. Evaluating the impact of mobile telephone technology on type 2 diabetic patients’ self-management: the NICHE pilot study. J Eval Clin Pract. 2008;14(3):465–9.
24. Hanauer DA, Wentzell K, Laffel N, Laffel LM. Computerized Automated Reminder Diabetes System (CARDS): e-mail and SMS cell phone text messaging reminders to support diabetes management. Diabetes Technol Ther. 2009;11(2):99–106.
25. Cho J-H, Lee H-C, Lim D-J, Kwon H-S, Yoon K-H. Mobile communication using a mobile phone with a glucometer for glucose control in Type 2 patients with diabetes: as effective as an Internet-based glucose monitoring system. J Telemed Telecare. 2009;15(2):77–82.
26. Newton KH, Wiltshire EJ, Elley CR. Pedometers and text messaging to increase physical activity: Randomized controlled trial of adolescents with type 1 diabetes. Diabetes Care. 2009;32(5):813–5.
27. Yoo HJ, Park MS, Kim TN, Yang SJ, Cho GJ, Hwang TG, et al. A Ubiquitous Chronic Disease Care system using cellular phones and the internet. Diabet Med. 2009;26(6):628–35.
28. Rodríguez-Idígoras MI, Sepúlveda-Muñoz J, Sánchez-Garrido-Escudero R, Martínez-González JL, Escolar-Castelló JL, Paniagua-Gómez IM, et al. Telemedicine influence on the follow-up of type 2 diabetes patients. Diabetes Technol Ther. 2009;11(7):431–7.
29. Istepanian RSH, Zitouni K, Harry D, Moutosammy N, Sungoor A, Tang B, et al. Evaluation of a mobile phone telemonitoring system for glycaemic control in patients with diabetes. J Telemed Telecare. 2009;15(3):125–8.
30. Rossi MCE, Nicolucci A, Di Bartolo P, Bruttomesso D, Girelli A, Ampudia FJ, et al. Diabetes Interactive Diary: a new telemedicine system enabling flexible diet and insulin therapy while improving quality of life: an open-label, international, multicenter, randomized study. Diabetes Care. 2010 Jan;33(1):109–15.
31. Noh J-H, Cho Y-J, Nam H-W, Kim J-H, Kim D-J, Yoo H-S, et al. Web-Based Comprehensive Information System for Self-Management of Diabetes Mellitus. Diabetes Technol Ther. 2010 May;12(5):333–7.
32. Kim CS, Park SY, Kang JG, Lee SJ, Ihm SH, Choi MG, et al. Insulin Dose Titration System in Diabetes Patients Using a Short Messaging Service Automatically Produced by a Knowledge Matrix. Diabetes Technol Ther. 2010;12(8):663–9.
33. Lim S, Kang SM, Shin H, Lee HJ, Yoon JW, Yu SH, et al. Improved glycemic control without hypoglycemia in elderly diabetic patients using the ubiquitous healthcare service, a new medical information system. Diabetes Care. 2011;34(2):308–13.
34. Quinn CC, Shardell MD, Terrin ML, Barr EA, Ballew SH, Gruber-Baldini AL. Cluster-Randomized Trial of a Mobile Phone Personalized Behavioral Intervention for Blood Glucose Control. Diabetes Care. 2011;34(9):1934–42.
35. Shetty AS, Chamukuttan S, Nanditha A, Raj RKC, Ramachandran A. Reinforcement of adherence to prescription recommendations in Asian Indian diabetes patients using short message service (SMS)--a pilot study. J Assoc Physicians India. 2011;59:711–4.
36. Goodarzi M, Ebrahimzadeh I, Rabi A, Saedipoor B, Jafarabadi MA. Impact of distance education via mobile phone text messaging on knowledge, attitude, practice and self efficacy of patients with type 2 diabetes mellitus in Iran. J Diabetes Metab Disord. 2012;11(1):10.
37. Arora S, Peters AL, Burner E, Lam CN, Menchine M. Trial to examine text message-based mHealth in emergency department patients with diabetes (TExT-MED): a randomized controlled trial. Ann Emerg Med. 2014;63(6):745–54.e6.

**Observational, non-randomized controlled, and uncontrolled trials**

1. Durso SC, Wendel I, Letzt AM, Lefkowitz J, Kaseman DF, Seifert RF. Older adults using cellular telephones for diabetes management: a pilot study. Medsurg Nurs. 2003;12(5):313–7.
2. Ferrer-Roca O, Cardenas A, Diaz-Cardama A, Pulido P. Mobile phone text messaging in the management of diabetes. J Telemed Telecare. 2004 Oct 1;10(5):282–5.
3. Vähätalo M, Virtamo H, Viikari J, Rönnemaa T. Cellular phone transferred self blood glucose monitoring: prerequisites for positive outcome. Pract Diabetes Int. 2004;21(5):192–4.
4. Kwon H-S, Cho J-H, Kim H-S, Lee J-H, Song B-R, Oh J-A, et al. Development of web-based diabetic patient management system using short message service (SMS). Diabetes Res Clin Pract. 2004;66 Suppl 1:S133-7.
5. Aoki N, Ohta S, Okada T, Oishi M, Fukui T. INSULOT: A cellular phone-based edutainment learning tool for children with type 1 diabetes. Diabetes Care. 2005;28(3):760.
6. Kim HS, Yoo YS, Shim HS. Effects of an Internet-based intervention on plasma glucose levels in patients with type 2 diabetes. J Nurs Care Qual. 2005;20(4):335–40.
7. Gammon D, Årsand E, Walseth OA, Andersson N, Jenssen M, Taylor T. Parent-child interaction using a mobile and wireless system for blood glucose monitoring. J Med Internet Res. 2005;7(5).
8. Wangberg SC, Årsand E, Andersson N. Diabetes education via mobile text messaging. J Telemed Telecare. 2006;12(5):55–6.
9. Kim H-S, Kim N-C, Ahn S-H. Impact of a nurse short message service intervention for patients with diabetes. J Nurs Care Qual. 2006;21(3):266–71.
10. Carroll AE, Marrero DG, Downs SM. The HealthPia GlucoPack ^TM^ Diabetes Phone: A Usability Study. Diabetes Technol Ther. 2007;9(2):158–64.
11. Kollmann A, Riedl M, Kastner P, Schreier G, Ludvik B. Feasibility of a mobile phone-based data service for functional insulin treatment of type 1 diabetes mellitus patients. J Med Internet Res. 2007;9(5):e36.
12. Katz DL, Nordwall B. Novel Interactive Cell-Phone Technology for Health Enhancement. J Diabetes Sci Technol. 2008;2(1):147–53.
13. Arsand E, Tufano JT, Ralston JD, Hjortdahl P. Designing mobile dietary management support technologies for people with diabetes. J Telemed Telecare. 2008;14(7):329–32.
14. Franklin VL, Greene A, Waller A, Greene SA, Pagliari C. Patients’ Engagement With “Sweet Talk” – A Text Messaging Support System for Young People With Diabetes. J Med Internet Res. 2008;10(2):e20.
15. Rossi MCE, Nicolucci A, Pellegrini F, Bruttomesso D, Bartolo P Di, Marelli G, et al. Interactive diary for diabetes: A useful and easy-to-use new telemedicine system to support the decision-making process in type 1 diabetes. Diabetes Technol Ther. 2009;11(1):19–24.
16. Turner J, Larsen M, Tarassenko L, Neil A, Farmer A. Implementation of telehealth support for patients with type 2 diabetes using insulin treatment: an exploratory study. Inform Prim Care. 2009;17(1):47–53.
17. Curran K, Nichols E, Xie E, Harper R. An Intensive Insulinotherapy Mobile Phone Application Built on Artificial Intelligence Techniques. J Diabetes Sci Technol. 2010;4(1):209–20.
18. Larsen ME, Turner J, Farmer A, Neil A, Tarassenko L. Telemedicine-supported insulin optimisation in primary care. J Telemed Telecare. 2010;16(8):433–40.
19. Hussein WI, Hasan K, Jaradat AA. Effectiveness of mobile phone short message service on diabetes mellitus management; the SMS-DM study. Diabetes Res Clin Pract. 2011;94(1):e24-6.
20. Mulvaney SA, Anders S, Smith AK, Pittel EJ, Johnson KB. A pilot test of a tailored mobile and web-based diabetes messaging system for adolescents. Journal of Telemedicine and Telecare. 2012;18(2):115–8.
21. Frøisland DH, Arsand E, Skårderud F. Improving diabetes care for young people with type 1 diabetes through visual learning on mobile phones: mixed-methods study. J Med Internet Res. 2012;14:e111.
